# Establishing a sustainable training programme in anaesthesia in Ethiopia

**DOI:** 10.1111/aas.14106

**Published:** 2022-07-21

**Authors:** Gunhild Holmaas, Ananya Abate, Assefu Woldetsadik, Olav Hevrøy

**Affiliations:** ^1^ Department of Surgical Services Haukeland University Hospital Bergen Norway; ^2^ Department of Anesthesiology Addis Ababa University, College of Health Sciences, School of Medicine Addis Ababa Ethiopia; ^3^ Ras Desta Damtew Memorial Hospital Addis Ababa Ethiopia

**Keywords:** Ethiopia, low‐income countries, postgraduate anaesthesia education, sustainability

## Abstract

**Background:**

Lack of qualified staff is a major hindrance for quality and safety improvements in anaesthesia and critical care in many low‐income countries. Support in specialist training may enhance perioperative treatment and have a positive downstream impact on other hospital services, which may improve the overall standard of care.

**Methods:**

Between 2011 and 2019, consultant anaesthetists from Haukeland University Hospital in Norway supported a postgraduate anaesthesia‐training programme at Addis Ababa University/Tikur Anbessa Specialised Hospital in Ethiopia. The aim of the programme was to build a self‐sustainable work force of anaesthetists across the country who could perform high quality anaesthesia within the confinement of limited local resources. Over the course of 10 years, an almost continuous rotation of experienced anaesthetists and intensivists assisted training of Ethiopian residents in anaesthesia and critical care. Local specialists organised the programme; however, external support was necessary during this period to establish a sustainable training programme.

**Results:**

Since the programme's commencement at Addis Ababa University in 2011, 159 residents have entered the programme and 71 have graduated. As the number of qualified anaesthetists increased, Ethiopian specialists gradually obtained responsibility for the programme. Candidates are recruited from various regions and from neighbouring countries. Five other Ethiopian training sites have been established. To date (May 2022), 112 residents have completed their training in Ethiopia, and 195 residents expect to graduate within 3 years.

**Conclusion:**

Nearly 11 years after establishment of the programme, locally trained highly qualified anaesthetists work in Ethiopia's major hospitals throughout the country.

**Editorial Comment**

This article describes how an educational program for anaesthetists was successfully implemented over a 10‐year period in Ethiopia. The authors emphasise the importance of respecting local culture and local resources, and to be aware of problems with modern equipment which may be dependent on local spare parts and maintainance. They also underline that as much as possible of the training should take place locally and not in a high‐income country, in order to avoid local practitioners leaving their country.

## 1 INTRODUCTION

Improvements in the safety and quality of anaesthetics and critical care is urgently needed in most low‐income countries (LIC).^1^, ^2^ A major challenge is the deficiency of trained anaesthesia staff, even in teaching hospitals. Investment in postgraduate training programmes in LIC should be a major priority.^3^, ^4^, ^5^, ^6^

The number of qualified anaesthetists and critical care physicians is critically low and often completely deficient in a majority of LIC.^4^, ^6^ Frequently, non‐physician anaesthesia (NPA) providers work alone without any support from consultants, and too often they are urged to handle cases beyond their qualifications.^7^, ^8^ The number of complications and deaths related to inappropriate care remains high.^9^, ^10^, ^11^, ^12^, ^13^, ^14^, ^15^, ^16^ In many hospitals, personnel without the appropriate competence also care for the critically ill.^15^

Improvement within anaesthesia safety, perioperative management and critical care quality may reduce mortality and have a beneficial impact on other aspects of health care as well, like infection control, emergency medicine and pain management.^15^, ^16^

Anaesthesia and critical care medicine in high‐income countries (HIC) has become increasingly dependent on complex equipment and expensive drugs. These high‐cost standards are beyond reach in many LIC.^16^, ^17^, ^18^ If basic equipment and essential drugs are available according to WHO–WFSA standards, adequately educated local professionals may establish routines for high quality treatment for a majority of patients.^17^, ^18^ However, building competence and establishing a staff of local well‐qualified experts requires enduring long‐lasting external commitment.^19^, ^20^, ^21^

Random efforts at competence building and uncritical transfer of standards from HIC without considering local constraints, often fail to improve training quality.^6^, ^22^, ^23^

Local training should include building a culture of rational decision‐making, reasoning and the ability to practice knowledge in accordance with local resources and culture. In most low‐resource settings, anaesthetists will be involved in emergency care, hence integrating anaesthesia and critical care in the same programme, as in Scandinavia, may be a coherent strategy.^24^, ^25^, ^26^

The authors have no conflicts of interest. This is a report and evaluation of a teaching project using official numbers of residents and graduates. We, therefore, consider institutional review board approval not necessary.

## 2 METHODS

Since 2011, consultant anaesthetists from Haukeland University Hospital (HUH)/University of Bergen (UoB) in Norway have been supporting a postgraduate training programme in anaesthesia at Tikur Anbessa Specialised Hospital (TASH)/University of Addis Ababa (AAU) in the capital of Ethiopia.

### 2.1 Aim of the project

The aim of the project was to assist Ethiopian colleagues in establishing a sustainable training programme for residents in anaesthetics in order to build an increasing qualified workforce within the country. To achieve this, recruitment of candidates from both major cities and more remote areas in different regions of the country was essential, and strategies to avoid brain drain imperative.^20^ The training should therefore take place in Ethiopia, and any fellowships abroad should occur in other LIC or in HIC for a limited period.

### 2.2 Background

Planning of the programme started in 2009. At that time, 11 consultant anaesthetists were working in Ethiopia, a country with then 96 million inhabitants. Most of the anaesthetists were educated in an AAU training programme established in 1990 and some in Eastern Europe during the communist period. In addition, some private external hospitals and charities had occasionally sent visiting consultants who assisted in the training of local physicians. Only four consultant anaesthetists were employed at TASH, the major teaching hospital in the country. Anaesthesia providers were mainly nurse anaesthetists with 4 years training. The local consultant anaesthetists were heavily overloaded and frustrated over lack of support from health authorities. The standard of anaesthesia at the hospital was poor, a limited number of drugs were available, and most of the equipment in use were external donations. Monitors were scarce, and many of the anaesthetic machines were not working properly. The intensive care unit (ICU) lacked basic equipment, and the few ventilators and monitors available were outdated.

### 2.3 Programme planning

In 2009, two new residents had entered the on‐going local anaesthesia‐training programme, the first of their kind in 7 years. Our first task was to fund a 3‐month anaesthesia‐ and intensive care fellowship in India for both of them.

In the following 2 years, we had regular meetings with the local stakeholders, colleagues and representatives from the hospital‐ and university management, discussing the programme and elaborating on what kind of collaboration and support they wanted and needed. A partnership agreement between the medical faculties and hospitals on both sides was signed.

Two of the staff anaesthesiologists at TASH visited Bergen, Norway where they had meetings with all partners and participants. A new curriculum for the programme was discussed and planned. It was emphasised that the curriculum should be in accordance with Ethiopian standards for postgraduate training and not copied from outside universities. Focus was placed on ensuring a cost‐effective structure in a low‐cost setting by introducing the Scandinavian four pillars of knowledge within the specialty structure (anaesthetics, critical care, pain management and emergency medicine).

The Medical Faculty at AAU later approved the curriculum and the programme. Due to new requirements and standards, several curriculum revisions were performed during the course of the programme.

### 2.4 Establishing the training programme

The new programme started in 2011 with the enrolment of five residents, four female and one male candidate who had recently graduated from the AAU Medical Faculty. The original plan was to enter four to five new candidates in the programme each year, which was considered suitable, given the local resources. However, the number of new residents was entirely decided by the health authorities and anaesthesia had a low status in the hospitals. Due to an increasing focus on safety and quality of perioperative care,^27^, ^28^ the health authorities gradually recognised the need for anaesthetists in the hospitals. The number of candidates was increased, and slowly acceptance and status of the specialty augmented.

During the period from 2011 until 2019, 12 Norwegian anaesthesia consultants supported the programme. Some had prior experience from Ethiopian hospitals, and one spoke the major local language, Amharic. During the first years, there was a close to continuous rotation of Norwegian anaesthetists staying for periods of 1–6 months in Addis Ababa, tutoring local residents in the theatres and the ICU. All participants were experienced consultants in various anaesthetic subspecialties including paediatric‐, neurosurgical‐, cardiothoracic‐ and obstetric anaesthesia and critical care. The idea was to provide the local residents an up‐to‐date education in the various fields of anaesthesia by giving lectures on specific topics, supervising and coaching the residents and contributing in practical skills training. Adjustment to a different culture and familiarisation to the local theatres and ICU standards was a challenge for all. Throughout this period of training, lectures on relevant topics were given regularly both to anaesthetic‐ and surgical residents who had training in anaesthesia and critical care incorporated in their curriculum. All communication and teaching was in English, the lingua franca at the university hospital.

In agreement with the local authorities, the majority of the training took place locally to prevent brain drain and to encourage development of treatment strategies appropriate to the low‐cost setting of the region. The first group of residents, however, were sponsored for a 2‐week visit to Norway for both motivational purposes and to get the opportunity to observe the Norwegian healthcare system.

### 2.5 Recruiting candidates

**FIGURE 1:**
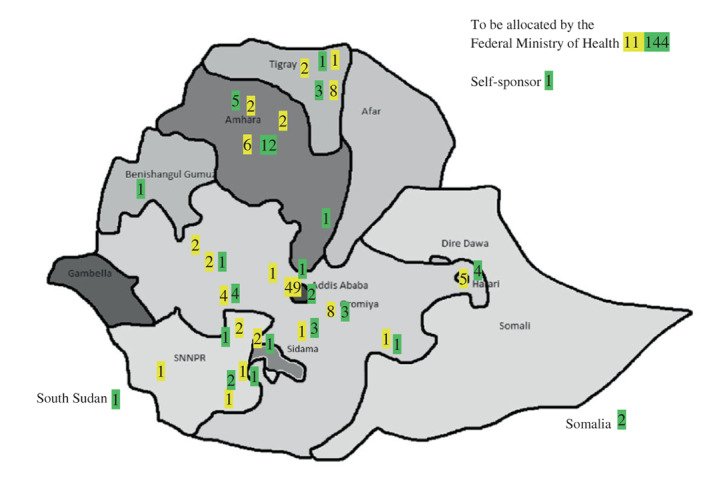
Graduated specialists in anaesthesia (yellow) and residents in training (green) in Ethiopia May 2022. Geographical distribution of funding countries, hospitals and regional states or chartered cities. The Federal Ministry of Health will allocate the remaining 144 residents after graduation

During the first years, all of the residents were from Addis Ababa. However, since 2014 an increasing proportion of applicants come from regional hospitals in different parts of the country (Table 1 and Figure 1). The residents receive salary from their local hospitals during the training period, and are obliged to perform 3–6 years of compulsory service at those hospitals after graduation.

**TABLE 1:** Location of compulsory service after anaesthesia graduation in Ethiopia 2011–2025: Number of anaesthetists funded by the Federal Ministry of Health (MOH), hospitals and regional health bureaus in Ethiopian regional states or chartered cities and neighbouring countries

Year of graduation	2011	2014	2015	2017	2018	2019	2020	2021	2022	2024	2025	Total
Allocated by MOH								11	23	44	77	155
Self‐sponsored											1	1
Addis Ababa	2	5	1	11	6	10	6	8				51
Oromia				2	2	3	2	10	10	1	2	32
Amhara					1	3	4	2	6	10	2	28
Tigray						3	4	4	2	2		15
SNNPR							2	3		3	2	10
Sidama						1	1					2
Harari						2	3			4		9
Benshangul Gumuz									1			1
South Sudan										1		1
Somalia									1	1		2
Total number of anaesthetists	2	5	1	13	9	22	22	38	43	68	84	307

*Note*: Year of performed or scheduled graduation.

Abbreviation: SNNPR, Southern nations, nationalities and peoples region.

Since 2015, more residency programmes have been established and launched in other regional hospitals and universities (Table 2). Presently, specialists and residents in anaesthesia represent 7 out of 10 regional states in addition to the capital, and even applicants from Somalia and South Sudan get their training in Ethiopian university hospitals. Since 2018, several of the residents have obtained sponsorship from the Federal Ministry of Health (MOH), which allocate the new specialists according to their priority when they graduate. This measure ensures that the number of qualified anaesthetists will gradually increase in the whole region, not only in the city of Addis Ababa.

**TABLE 2:** The Ethiopian anaesthesia education programme at different training sites 2009–2022: Number of graduates, residents, home cities and funding hospitals

Year of graduation	2011	2014	2015	2017	2018	2019	2020	2021	2022	2024	2025	Total
Graduation	2	5	1	13	9	22	22	38				112
Scheduled graduation									43	68	84	195
Training sites												
1 TASH	2	5	1	11	7	18	16	11	23	31	34	159
2 JUTH				2	2		6	9	11	10	9	49
3 SPHMMC						4		18	9	16	21	68
4 TGSH										7	6	13
5 HFSH										4	9	13
6 HURH											5	5
Geographical distribution												
Home cities	1	1	1	3	3	9	9	13	6	9	5	24
Funding hospitals	1	1	1	6	5	12	13	13	7	11	5	31

*Note*: Year of performed or scheduled graduation. 1—Tikur Anbessa Specialised Hospital/Addis Ababa University, Addis Ababa. 2—Jimma University Teaching Hospital, Jimma, Oromia. 3—St. Paul's Hospital Millennium Medical College, Addis Ababa. 4—Tibebe Gion Specialised Hospital/Bahirdar University, Bahirdar, Amhara. 5—Hiwot Fana Specialised Hospital/Haromaya University, Harar, Harari. 6—Hawassa University and Referral Hospital, Hawassa, SNNPR and Sidama.

### 2.6 Critical care medicine

To fulfil the idea of including critical care medicine in the programme, it was necessary to improve the technical standard of the ICU at TASH.^24^ We, therefore, applied for and received a donation from a private sponsor to better equip the newly renovated paediatric and adult medical and surgical ICU. The funding enabled us to purchase monitors and ventilators, and we invested great efforts in establishing maintenance agreements and secure spare part delivery from local Ethiopian companies and distributors. Over the last years, a new generation of ventilators and monitors purchased by Ethiopian health authorities have been installed. However, equipment maintenance and spare part delivery is still a problem that needs to be addressed.

To improve ICU standards, ICU nurse training was essential. Having a close collaboration with a neurosurgical training programme sponsoring nurses from Norway to work in Ethiopia, we obtained indispensable support from experienced ICU nurses who worked alongside local nurses.^29^ Treatment protocols for several common diagnoses were implemented, and nurses and doctors were trained together as a team to handle emergency cases. A nutrition formula for nasogastric tube feeding for ICU patients based on ingredients available on the local market was introduced. In addition, a new ICU observation sheet was developed and approved by the hospital management and is now in use both at TASH and in several other Ethiopian ICUs. We also launched the BASIC programme; a Hong Kong based non‐profit critical care coursing system, and so far, four courses and one trainer course have been launched.^30^, ^31^ One of the Ethiopian anaesthesia consultants is now course facilitator.

### 2.7 Curriculum, training and exams

The curriculum consists of a 3‐year programme, starting with an introduction period with lectures on basic sciences and training in fundamental skills.^21^ The residents then start their practical training in the theatres supervised by senior colleagues. During the first year, they rotate with short intervals between anaesthetic services within different surgical specialties. In the second and third years, they continue their rotation with longer intervals, with the candidates gaining more responsibility and exposure to procedures and further training in practical skills. Critical care training is included, and the residents take part in on‐call duties both at the ICU and in the theatres.

The hospital is equipped with skill‐labs for manikin‐resuscitation and ‐intubation.^12^, ^19^ The residents have used bananas as skin substitutes to practice insertion of central venous lines and epidural catheters. We have brought equipment for difficult airway handling like bronchoscopes and video laryngoscopes, and regular training sessions in such skills are mandatory. Due to donations from abroad and intensive training, most of the consultant anaesthetists now perform simple echocardiography techniques.

After the first and second year of training, the residents have to pass an in‐term exam in order to continue their training. To finish the programme and get the specialty certificate in anaesthesia approved by the AAU, the residents have to pass a final exam including both oral and written tests. A mandatory research project is also included.

### 2.8 Infrastructure and hospital services

Although the technical standard has improved vastly over the last decade, the basic infrastructure and services in the hospital are still challenging. A limited number of laboratory tests are available, and radiology services are not always accessible. The most bothersome experience when establishing ICU facilities has been the lack of blood gas analyses, which is a necessity in critical care medicine. Being aware of this, the government purchased a large number of advanced blood gas machines from ABL. They unfortunately did not ensure the delivery of reagents necessary to run the analysers. The technical skills to maintain the machines were also lacking. Consequently, these expensive machines were used until they ran out of reagents and are now left to waste in storage rooms. This is a common example of the lack of collaboration between health authorities, local staff and delivery companies. It is tempting to challenge businesses delivering expensive technical equipment to hospitals in LIC, to be obliged to guarantee that supplies and knowledge to run the machines are in place.

### 2.9 Disposables and drugs

One of the difficulties faced during the whole period has been to accomplish a stable supply of basic drugs and consumables for anaesthesia and intensive care treatment. The drugs available are limited. There have been some improvements during the decade; however, stable and foreseeable drug supply is still missing. Solving the problem requires political involvement at the government level.^28^The same problem is relevant for most disposable stocks. The lack of efficient purchase systems for drugs and consumables is frustrating to all involved, and contributes to poor quality of care.

### 2.10 Partners

A group of anaesthetists from Toronto University Hospital joined the programme in 2011. They organised their visits as groups of two to three specialists giving lectures and practical training for 2–3 weeks at a time. From 2017, anaesthetists from the Canadian Association of Anesthesiologists International Education Foundation replaced the Toronto group. The contribution from Canada has been a major condition for implementation of the programme.

Likewise, a dedicated group of anaesthetists from Swedish Medical Center in Seattle were also active supporting anaesthesia and surgery at TASH in the same period. In addition to teaching, they contributed with medical equipment supply. They equipped the first post‐surgery recovery unit at TASH in 2009.

In 2013, the Government of Ethiopia launched a programme with rapid expansion of teaching institutions for NPA consisting of the already existing 4‐year bachelor's degree for high school graduates, a 3‐year baccalaureate training for nurses and a 2‐year post‐bachelor master's degree. In addition, a 1‐year level‐V anaesthetic training for nurses was established tailored to provide anaesthetic care in caesarean section, laparotomy and fracture management.^32^ Anaesthetist nurses from Johns Hopkins University in Baltimore supported the anaesthetist master's degree training at TASH. There was no direct cooperation between the two programmes, however we met regularly and supported each other when required.

### 2.11 Financing

None of the Norwegian anaesthetists participating in the programme received any salary or economic compensation. They used their sabbatical periods in Addis Ababa. Funds from Department of International Collaboration at HUH covered travel expenses, accommodation and teaching expenditure. For the first 2 years, there was also an additional support from the Norwegian Agency for Development Cooperation through the UoB. Donations from a private sponsor covered technical equipment budgets at the ICU. No direct financial support was offered to TASH.

Total spending during 10 years was $128.207 to run the Norwegian part of the programme and $266.796 for equipment (June 2021 currency exchange rates).

## 3 RESULTS

Table 1 and Figure 1 show the number and geographical distribution of anaesthetists educated in Ethiopia since 2011. The progress of the programme at AAU and the five other established training sites are listed in Table 2. Ayder Referral Hospital/Mekele University in the Tigrayan capital also enrolled anaesthetic residents in 2021, but due to the war in Tigray, they were not able to start.

Since 2011, 71 new anaesthetists have finished their training and 88 expect to graduate within 3 years at TASH.

In addition to the total number of anaesthetists, probably the most constructive result of the programme is the distribution of sponsoring hospitals in 7 out of 10 regional states in addition to the capital. The counter brain‐drain strategy of implementing the training locally and the commitment of compulsory service at the funding hospitals after graduation, has been successful. Only 3 out of 71 specialists educated at AAU have left the country, and one of them moved to Somaliland, an area in desperate need for anaesthesia service.

## 4 DISCUSSION

This report shows that it is possible to establish a specialist‐training programme in anaesthetics and critical care medicine in a LIC with limited resources. Considering the number of anaesthetists who have completed their training, the programme has been a success due to long‐term continuous support and close collaboration with local partners as the key elements (Tables 1 and 2).

Local experts have been responsible for managing the programme; however, external help to establish the training was significant in the beginning. In one decade, the number of anaesthetists has increased multiple times mainly due to the programme, and anaesthesia has become gradually more recognised as a medical specialty. Ethiopian legislation affirms that anaesthetists should be present at all comprehensive specialised hospitals.^27^, ^28^ Although not fulfilled, anaesthesia and basic critical care medicine has gained acceptance as an important part of the health service.^28^

Unfortunately, we have no data documenting the effect of the programme on frequency of serious incidents, morbidity and mortality. Lack of reliable data and appropriate severity scoring tools made it difficult to compare quality of services before and after implementation. Since 2011, surgery has evolved dramatically with several new surgical specialties being introduced, making comparison even more difficult.^22^, ^29^, ^33^ In our experience, the increase in competence and upgraded training soon improved standards of care and augmented quality of service. In general, quality of anaesthetic services highly correlates with perioperative deaths and morbidity.^13^, ^14^, ^15^

Since 2015, the number of residents has increased faster than predicted due to the increased standing of education in general and medical postgraduate training in particular. Currently, the local staff manage the training and organise the teaching programme with little outside support; however, they are still pleased to have some backing from external experts.^14^, ^15^, ^18^, ^19^, ^20^

It has been a major concern that the fast‐growing number of new residents might jeopardise the quality of training. The number of theatres in each hospital is limited, and the follow‐up of individual resident is difficult when the number is increasing so fast. So far, the motivation and enthusiasm of the candidates are high, and this is probably compensating for shortness of teaching facilities, but an open discussion on quantity versus quality in training is necessary.

A particularly favourable result of the programme is that an increasing number of skilled anaesthetists now work in different parts of the country, and the standard of surgical care is improving in the country as a whole (Figure 1). The idea of recruiting residents from different regional hospitals has been successful and is now generally accepted. The obligatory service at the funding hospitals after graduation secure a widespread improvement of perioperative care. In addition to the increasing number of residents at AAU, several other major hospitals and regional universities have established their own training programmes, and the number of specialists will probably continue to rise in the coming years (Table 2).

An important reason for the success of the programme has been the collaboration between external experts and local champions based on mutual trust. Even if the staff were small, they consisted of key personnel able to launch shared ideas. Giving the programme a local profile and securing a local ownership has been essential.^20^ External support has motivated, supported and strengthened the programme until the local affiliates were able to carry on themselves. We believe that this kind of self‐sustainable thinking is essential for success.

Lack of communication and trust between the local experts and the health authorities has been an obstacle for development, especially in the beginning. This is particularly obvious when it comes to procurement of new equipment, when big investments area made without involving the users. Now, the Ethiopian Society of Anesthesiologists Professional Association, founded in 2009, has increasing influence and works together with the Ethiopian Association of Anaesthetists and MOH to further develop the field of anaesthesia. Together their aim is to establish a common Ethiopian standard of quality and safety^27^, ^28^ and sufficient import and distribution of drugs, consumables, spare parts and equipment is one of their priorities.^28^

The poor standard and lack of basic equipment often overwhelm foreigners visiting hospitals and ICU facilities in LIC. Too often, this generates donations of equipment considered necessary; however often randomly collected and outdated.^34^, ^35^ This kind of support is well‐intentioned but often useless and sometimes harmful due to lack of follow‐up and training.^24^, ^25^, ^34^ Medical equipment in LIC should be easy to maintain and not dependent on specialised spare parts or consumables from abroad. Local medical engineers should be empowered to maintain equipment, and local healthcare workers should always be able to monitor basic vital functions on critically ill patients without much advanced technology.^12^, ^17^ We will endorse purchasing of new equipment including agreements on maintenance and supply.

Running a programme over a long period following an essential plan with common goals has been essential in our view. All local initiatives should be appreciated, and efforts to improve standard supported and encouraged. All training should be grounded on the principles of safety, quality and responsibility.^35^, ^36^, ^37^

Experts in different fields regularly visit Ethiopian hospitals and universities and want to give lectures and assistance to local healthcare workers. Often, teaching based on standards taken from HIC are not adjusted to the local needs. We have focused on developing local standards based on available resources.^7^, ^35^

Collaboration between universities and neighbouring countries to develop common teaching standards and programmes, exchange of ideas and experience in the same region and continent, should be encouraged.^19^, ^20^, ^21^ The need for assistance in health care in LIC might seem overwhelming, but we believe that focusing on competence building and establishing their own capable personnel in different areas of medicine is a way forward.

## 5 CONCLUSION

This project shows that it is possible to achieve success in capacity building of anaesthesia and basic critical care in a LIC even with a small‐scale programme and limited resources. The fast‐increasing number of trained anaesthetists qualified through this programme gives hope for gradual improvement of safety and quality of anaesthesia and perioperative care in the future. Having an adequate number of well‐trained experts who can set local standards, is a core requirement. To achieve good results it is fundamental to have long‐lasting commitments and to encourage local efforts and leadership. Local needs and traditions should be valued, and building self‐confidence in the ability to create local competence and labour is important. We believe that strong leadership is required to ensure development of robust systems for high quality of perioperative care, which will in turn improve patients' safety.

## AUTHOR CONTRIBUTIONS

Aman Edao, Yemane Ayele, Samrawit Tassew and Denekew Assefa contributed with information regarding residents and specialists educated at Hawassa University and Referral Hospital, Jimma University Teaching Hospital, St. Paul's Hospital Millennium Medical College and Tibebe Gion Specialised Hospital, respectively. Heather Liegh Wieman contributed as English language consultant.

## CONFLICT OF INTEREST

The authors declare no conflict of interest.
